# Variation of parasitism patterns in bats during hibernation: the effect of host species, resources, health status, and hibernation period

**DOI:** 10.1007/s00436-016-5138-7

**Published:** 2016-05-30

**Authors:** Tomasz Postawa, Zoltan Nagy

**Affiliations:** 1 Institute of Systematics and Evolution of Animals, Polish Academy of Sciences, Sławkowska 17, 31-016 Kraków, Poland; 2 Foundation for School, Densusianu Str. 6/A, 400428 Cluj-Napoca, Romania

**Keywords:** Hemoglobin, Hibernation, Mouse-eared bats, Parasite infection, Resources

## Abstract

During critical periods of food shortage or variable climatic conditions, the choice of an appropriate host can increase the survival and reproductive performance of parasites. In turn, one of the unique adaptations to periodical food shortages is hibernation, which is often found among insectivorous bat species in the temperate zone. While hibernating, bats are completely defenseless against both predators and ectoparasites, their immune and endocrine systems are diminished, and survival is dependent on the accumulated fat reserves. Differences in the health status or in the rate of consumption of the resources might also explain species-specific differences in ectoparasite abundance, especially between closely related host species, such as the greater mouse-eared bat (*Myotis myotis*) and the lesser mouse-eared bat (*M. blythii*) during hibernation. In the present study, the abundance of two ecologically distinct (summer and winter) types of ectoparasites was examined in terms of its influence on the body condition and hemoglobin content of the two host species. The effects of demographic factors, such as host sex and age, were also investigated. Despite a similar pattern of deteriorating body condition and hemoglobin concentration, *M. myotis* was more parasitized than was *M. blythii*. The marked decrease in hemoglobin content in first-year females of both host species correlated with the highest parasite load and indicated a risk of anemia. At the intraspecific level, ectoparasite abundance was not correlated with body condition (resources), but it negatively affected hemoglobin content; however, this mostly concerned *M. blythii*, which had a lower parasite load. Therefore, it can be concluded that interspecific differences in ectoparasite abundance may result from parasites selecting the host species that is less sensitive to their activity. In turn, in summer ectoparasites, the preference for female hosts is probably attributable to the likelihood of reinfection rather than to an effect of host resources or health status. The absence of sex-based preferences in winter ectoparasites could be explained by equal host availability.

## Introduction

Non-random patterns of parasite abundance within a host species can be attributable to various extrinsic and intrinsic factors (Combes 2001). First, parasite infestation may depend on the host’s health status (Christe et al. 2003; Reckardt and Kerth 2009), sex (Morand et al. 2004; Krasnov et al. 2012) and age (Hawlena et al. 2005). The host’s susceptibility to parasitism is influenced both by its resources and the function of its immune system. Ectoparasites might choose hosts with low immunocompetence (Bize et al. 2008) or evolve strategies to avoid immunoreactions (Ribeiro 1995; Wikel 1999). Usually, the host’s immune function is associated with the stored energy reserves (Corbin et al. 2008). Host species exhibiting sex differences in terms of body condition often show concurrent sex-specific differences in parasite load. According to the vulnerable host hypothesis (Christe et al. 2000), the sex with an inferior body condition may be more heavily parasitized. Conversely, the well-fed host hypothesis explains a larger parasite load in one sex as resulting from better nutritional resources (Christe et al. 2003; Hawlena et al. 2005). Thus, there is still no consensus as to the causal relationships between the resources of hosts, or their benefits, and the abundance of ectoparasites. The individual resources or reserves of the hosts are often correlated with hematological parameters as hematocrit or hemoglobin concentration (Thomas et al. 2007). Fluctuations in the availability of food resources may affect energy reserves and immunocompetence, and thus increase susceptibility to parasitism. In fact, periods of environmental variation and resource limitation are critical for both hosts and ectoparasites (Rueesch et al. 2012). Unfavorable conditions and periods of increased mortality or inactivity should influence the selection of an appropriate host for parasites. Thus, choosing the right host can increase the probability of ectoparasite survival, reproduction, and/or reinfection (Krasnov et al. 2005a).

Hibernation, being a unique adaptation to periodical food shortages, is widespread among insectivorous temperate-zone bat species (Speakman and Thomas 2003). Prior to hibernation, bats mate around their winter shelters (Furmankiewicz and Górniak 2002; Parsons et al. 2003) and accumulate additional fat reserves to survive the winter (Kunz et al. 1998; Kokurewicz and Speakman 2006). During hibernation, the body temperature of the bats declines to approx. 1–2 °C above the ambient temperature and their metabolic rate decreases dramatically (Geiser 2004). Bat metabolism in hibernation is reduced, with activity occurring during periodic arousals from torpor (Park et al. 2000), which account for the consumption of the majority of the stored energy (Thomas et al. 1990). Therefore, hibernation is considered a critical period in the life of bats, especially juveniles, and high winter mortality has been observed (Horáček 1985; Tuttle 1976; Speakman and Thomas 2003; but also see Ruczyński et al. 2005; Turbill et al. 2011). Resource accumulation depends to some extent on the sex and age of the host—adult females have greater fat reserves and consume them more slowly than adult males and yearlings do (Koteja et al. 2001; Jonasson and Willis 2011). With the metabolic rate reduced during hibernation, erythropoiesis and hormone levels are diminished (Kawamoto 2003). Moreover, prolonged torpor compromises immunocompetence (Burton and Reichman 1999; Moore et al. 2011; Bouma et al. 2012), while grooming activity is constrained to short episodes during arousals, which makes bats almost defenseless against parasitic infections during hibernation.

Bats are typically infected by several highly specialized blood- or lymph-feeding groups of ectoparasites which can be divided into “summer” and “winter” parasites based on the time of their breeding (Dusbábek 1972). The reproduction of the summer ectoparasites: Spinturnicidae, Nycteribiidae, and Siphonaptera are correlated with their hosts’ reproduction; hence, they are most abundant during summertime and less so during the hibernation period (Hůrka 1964; Zahn and Rupp 2004; Lourenço and Palmeirim 2007). Conversely, the abundance of Macronyssidae and Ixodidae is the highest in winter, during host hibernation, and is correlated with the reproduction of parasites (Dusbábek 1972; Haitlinger 1978). The low temperature during the hibernation period should strongly reduce the food intake of the summer ectoparasites; however, they are often found to contain blood, which suggests that feeding continues through winter (Reisen et al. 1976). Ectoparasites are not very resistant to food scarcity: at a temperature of 8 °C, Nycteribiidae die after 5 days without food (Hůrka 1980) and Spinturnicidae after 3 days (Deunff and Beaucournu 1981), which may also indirectly confirm the hypothesis of winter feeding.

In contrast to most mammalian hosts, where males tend to have a significantly higher parasite prevalence and intensity than females do (Morand et al. 2004; Krasnov et al. 2005b), in temperate bat species, this trend is regularly reversed (Christe et al. 2007). Sex-biased parasitism in bats is explained by the larger female resources (Christe et al. 2003) or by strategies aimed at reinfection as a result of female sociality characterizing most bat species (Zahn and Rupp 2004; Reckardt and Kerth 2009). Furthermore, physiological differences between male and female hosts may be different in reproductive and non-reproductive periods, directly leading to seasonal patterns in sex-biased parasitism (Krasnov et al. 2012). Differences in parasite infection are also seen between adult and juvenile hosts and may be the result of differences in immune resistance (Christe et al. 2000), the efficiency of ectoparasite removal during grooming (Hawlena et al. 2007), or time of ectoparasite accumulation (Hawlena et al. 2006). Finally, although bat ectoparasites tend to be host-specific, some of them can utilize a variety of hosts with varying intensity (Marshall 1982). In the case of closely related host species infected with the same ectoparasite species, even small differences in morphology, ecology, or diet can lead to different susceptibilities to a particular parasite (Freeland 1983).

In this study, we examined whether the abundance of hematophagous ectoparasites was related to the hibernation period (early and late) and host condition (body condition index and hemoglobin concentration) in different sex and age classes of the two host bat species. We investigated two sibling bat species: the greater mouse-eared bat (*Myotis myotis* Borkhausen, 1797) and the lesser mouse-eared bat (*M. blythii* Tomes, 1857), which co-occur throughout the majority of their European range (Furman et al. 2014). In Central Europe, individual members of these species are found in winter shelters from mid-November, while more bats begin to hibernate in late November and early December. For wintering, they choose large underground sites with a stable microclimate (Nagel and Nagel 1991) and often form clusters of up to several hundred individuals (Güttinger et al. 2001). In *M. myotis*, the hibernation season extends for 4–5 months (Wojciechowski et al. 2007), and individual torpor bouts typically last from a 3 to 20 days (Harmata 1987). In the course of hibernation, large mouse-eared bats lose several percent of their body weight, mainly fat reserves, with males losing more weight than females do (Koteja et al. 2001). They leave their winter shelters in mid-April, although some individuals remain underground until May. In contrast to breeding colonies, mouse-eared bats of both sexes and all ages are present in winter aggregations (Koteja et al. 2001; Gazaryan 2007).

We investigated the effects of host demography and ecology on ectoparasite abundance during winter hibernation. First, we tested the hypothesis that ectoparasite abundance differs between species as well as between host age and sex groups. The second hypothesis was that ectoparasite abundance differs between hibernation periods. According to the third hypothesis, the condition parameters such as the body condition index (BCI) and hemoglobin concentration (Hg) are correlated with ectoparasite abundance. One can also expect different patterns of changes in the summer and winter ectoparasites due to the differences in their biology.

## Materials and methods

### Study area

The study was conducted in Peştera Apă din Valea Leşului (the Water Cave in the Lesului Valley; N 46.82472; E 22.55678, altitude: 823 a.s.l); Apuseni Mts., Romania. The cave was visited twice during the hibernation period: at the beginning (December 18–21, 2010) and end (April 11–14, 2011) of the hibernation period. The microclimate of the site is stable and the temperature in the part primarily inhabited by bats during winter varied from 3.3 to 6.6 °C (with a mean of 5.2 °C), with almost 100 % relative humidity (Szodoray-Parádi and Szántó 1998; Fejér and Moldovan 2013). Most large mouse-eared bats hibernated approx. 80–100 m from the entrance in a cave segment extending for approx. 150–200 m, and formed clusters of up to several hundred individuals. The cave constitutes one of the largest wintering shelters of large mouse-eared bats in Romania (Nagy and Postawa 2011), and during this study, 5471 *M. myotis*/*M. blythii* individuals were found there.

### Bat sampling and ectoparasite collection

Greater mouse-eared bats (*Myotis myotis*) and lesser mouse-eared bats (*M. blythii*) were collected only from mixed aggregations with more than 100 individuals per cluster. Both host species were available to a similar degree and hibernated under the same conditions. The bat species were preliminarily differentiated based on a small dark spot at the tip on the tragus (Dietz and von Helversen 2004), which is visible without disturbing the bats. After capture, bats (25–30 individuals per day) were immediately transported by car to the laboratory located about 15 min away from the cave. The animals were stored in a refrigerator at 6–8 °C and 100 % humidity and were taken out immediately before ectoparasite collection and measurements. The collected bats were first identified based on upper tooth row length (L C1–M3), which is the most characteristic difference between the two studied species (Furman et al. 2014). Juveniles (first-year individuals) and adults were distinguished on the basis of fur color, the presence/absence of a black spot, and the degree of tooth wear. For each bat, forearm length (digital caliper, accuracy 0.01 mm) and body mass (digital scale, accuracy 0.01 g) were measured. About 50 μL of blood was taken from the ventral vein of the uropatagium using a 0.3 × 13-mm ultrathin needle. A drop of blood was transferred by pipette to a URIT-12 hemoglobin meter strip (Hemo-Test Urit-12, Urit Medical Electronic co. LTD.), for quantitative measurement of total hemoglobin in peripheral blood (g/dL). To avoid bleeding after blood collection, cautery gel (Super Clot Gel, Synergy Labs) was applied to the puncture site to disinfect it and close the wound. In order to restore normal blood circulation, prior to blood sampling, the animals were kept for about 15 min at room temperature. Two parameters were applied as measures of bat condition: the body condition index (ratio of body mass to forearm length: Speakman and Racey 1986) as a proxy for the accumulated resources (Pearce et al. 2008) and hemoglobin concentration (g/dL), representing the physiological condition (Fair et al. 2007).

The fur, wing membranes, and ears were carefully examined for ectoparasites. A cotton bud with a drop of ethyl acetate was used for poisoning bat flies. Ectoparasites were collected from bats using tweezers and exhaustors and fixed in ethyl alcohol (95 %). Species were identified in the laboratory using a light microscope. Wing mites were classified following Dusbábek (1962) and Stanyukovich (1997), and bat flies were identified according to Theodor and Moscona (1954) and (Hůrka 1980). We also used a comparative collection of ectoparasites. Due to their large numbers, Macronyssidae were not determined at the species level; however, in large *Myotis* species, only two parasitic species may account for nearly 99 % of all the arthropods found (Dusbábek 1972; Haitlinger 1978). In total, 5325 ectoparasites were collected: *Spinturnix myoti* Kolenati 1856 (220 individuals), *Nycteribia latreillii* (Leach, 1817) (82), *N. vexata* Westwood 1835 (372), *Penicillidia dufourii* (Westwood, 1835) (147), Macronyssidae (4504), Ixodidae (3), and Siphonaptera (1). A total of 217 bats, 117 *M. myotis* and 100 *M. blythii*, were examined for BCI and ectoparasites and had blood samples taken. After all measurements were made and parasites collected, the bats were released in the cave near the place where they had been collected.

The field study was carried out under a license issued for the project LIFE08 NAT/RO/000504 by the competent Romanian authorities.

### Data analysis

We used mean abundance, which is the average number of ectoparasites for a given species per individual host (Bush et al. 1997), as the parasitological parameter. Since the distribution of ectoparasite abundance is usually skewed, we used a logarithmic transformation (log (*n* + 0.5)) in ectoparasite abundance analysis. The factors potentially influencing ectoparasitic abundance were assessed using a general linear model (GLM) with a Gaussian distribution and an identity link function. Mean abundance values are reported as the mean number of ectoparasites ± standard error (SE).

GLMs were constructed by fitting the explanatory variable: fixed factors (period of hibernation, host age, sex, and species) and covariates (the body condition index, hemoglobin concentration), which could potentially influence ectoparasite abundance. Separate GLMs were used to test species-specific differences in bat condition parameters and parasite load and to evaluate variation in each calculated ectoparasite abundance value, separately for the two investigated host species, three factors (host sex and age and hibernation period), and two covariates (body mass index and hemoglobin concentration).

A post hoc multiple-comparison least significance difference (LSD) test was carried out for parasite load, BCI and Hg between host sex/age classes for each period and species separately. The significance level used was *p* < 0.05. Statistical analyses were performed using STATISTICA software version 6.0 (StatSoft Inc., Tulsa, OK, USA) and R (R Development Core Team 2011).

## Results

### Physiological condition of the hosts

**Fig. 1:**
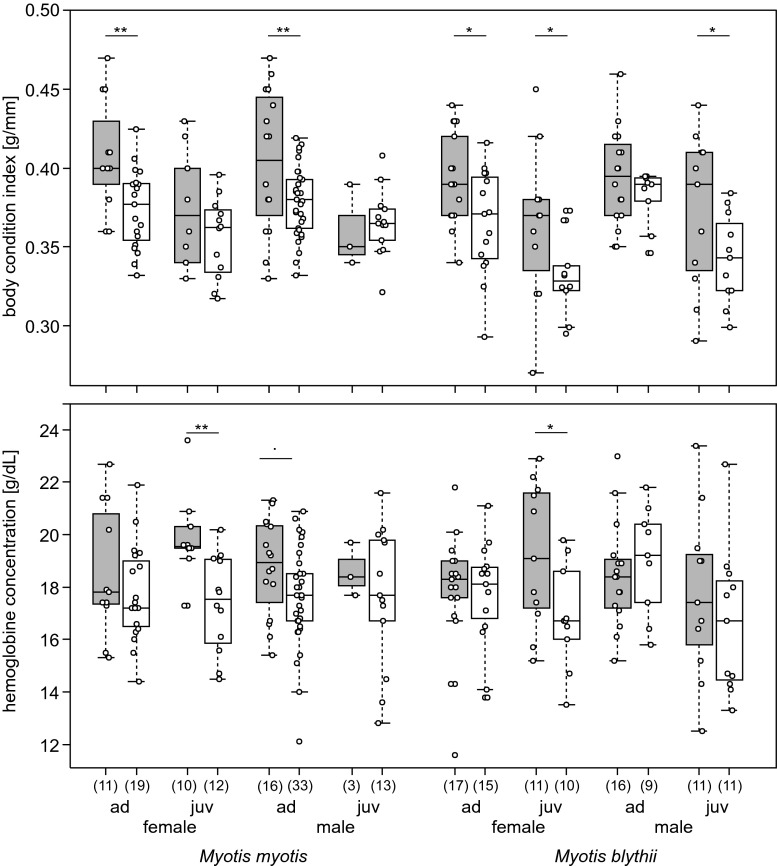
Body condition index [g/mm] (**a**) and hemoglobin concentration [g/dL] (**b**) in the two host species: *Myotis myotis* and *Myotis blythii*, in sex and age classes, in early (December) and late (April) hibernation periods. *Ad* adult, *juv* juvenile. *Numbers in parentheses*—sample sizes. Significant differences between early and late hibernation within each sex and age classes of host species (post hoc LSD) are marked with an *asterisk*: * *p* < 0.05, ** *p* < 0.01, *** *p* < 0.001

The GLM revealed significant differences in the body condition index and hemoglobin concentration in both host species, but with distinct patterns for *M. myotis* and *M. blythii*. The body condition index of *M. myotis* was significantly higher in December than in April (*p* = 0.006, Table 1), adults were heavier than juveniles were (*p* < 0.0001, Table 1), and a nearly significant interaction was found between period and age (*p* = 0.091, Table 1): during hibernation, BCI decreased more in adult bats than in juveniles; no significant interactions were found (Table 1). Post hoc (LSD) analysis restricted to differences between periods revealed a significant decline of BCI for adults: males and females (Fig. 1). In the other host species (*M. blythii*), the body condition index was also significantly higher in December than in April (*p* = 0.0003, Table 1) and adults were heavier than juveniles were (*p* < 0.0001, Table 1), but no significant interactions were found (Table 1). Post hoc (LSD) analysis of differences between periods showed a significant decline of BCI during hibernation for adult and juvenile females and for juvenile males (Fig. 1).

**Table 1:** Results of GLM analysis for the body condition index (BCI) and hemoglobin concentration (Hg) in two host species: *M. myotis* (*N* = 100) and *M. blythii* (*N* = 117) as a function of period (December, April), sex (male, female), and age (juvenile, adult)

	*Myotis myotis*		*Myotis blythii*		*Myotis myotis*		*Myotis blythii*	
	Body condition index [g/mm]				Hemoglobin concentration [g/dL]			
	*F*	*p*	*F*	*p*	*F*	*p*	*F*	*p*
Intercept	12982.8	<0.0001	11097.0	<0.0001	6434.0	<0.0001	5438.6	<0.0001
Period	7.75	0.006	14.4	0.0003	9.08	0.0032	2.30	0.133
Sex	0.04	0.84	1.60	0.208	0.24	0.624	0.01	0.946
Age	17.0	0.0001	20.1	<0.0001	0.27	0.607	1.77	0.186
Period × sex	1.64	0.204	0.52	0.471	0.51	0.475	1.04	0.312
Period × age	2.91	0.091	0.65	0.422	0.57	0.454	3.85	0.053
Sex × age	0.04	0.848	0.04	0.833	0.34	0.560	3.32	0.072
Period × sex × age	0.28	0.596	0.27	0.603	1.08	0.301	0.109	0.742

Hemoglobin concentration in *M. myotis* significantly differed only between periods, that is, in April, it was lower than in December (*p* = 0.003, Table 1). No significant differences were found among other factors and their interactions (Table 1). Post hoc (LSD) analysis of differences between periods revealed a significant decline of hemoglobin in juvenile females and a nearly significant one in adult males (Fig. 1). In turn, in *M. blythii*, the seasonal effect was not significant, but near-significant differences were found for two interactions: between period and age (hemoglobin concentration deteriorated in juveniles, but not adults, during hibernation, *p* = 0.053, Table 1) and between sex and age (in both sexes, adults and juveniles differed in terms of hemoglobin concentrations, *p* = 0.072, Table 1). Post hoc (LSD) analysis revealed differences between December and April with a significant decline in hemoglobin concentration only in juvenile females (Fig 1).

### Parasites

#### Host species-specific differences in ectoparasite abundance

**Fig. 2:**
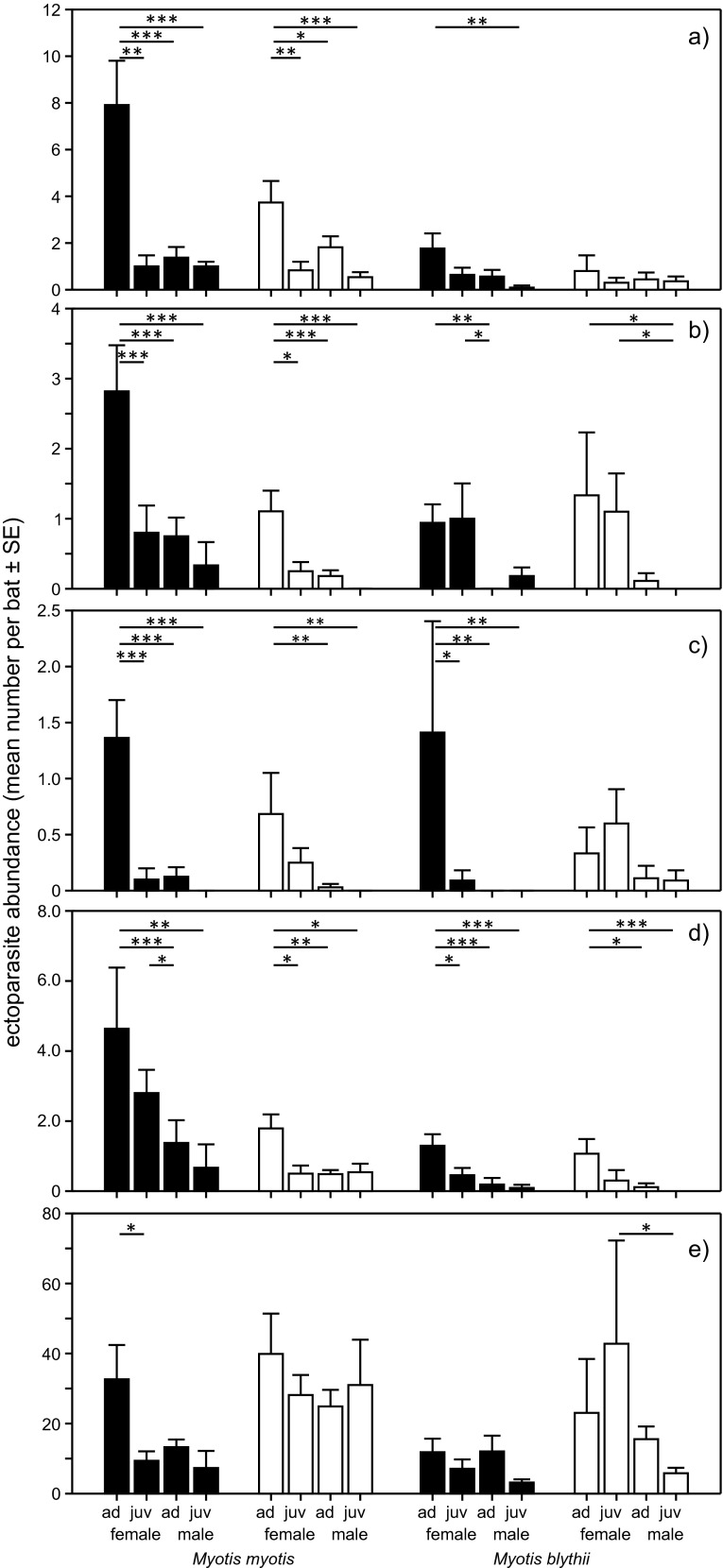
Ectoparasite abundance (mean number per bat ± SE) of **a** N. vexata, **b** P. dufourii, **c** N. latreillii, **d** S. myoti, **e** Macronyssidae, in sex and age classes of two host species: *M. myotis* and *M. blythii* during early—December (*black bars*) and late—April (*white bars*) hibernation period. The *lines above bars* indicate significant differences between classes (post hoc Tukey test): * *p* < 0.05, ** *p* < 0.01, *** *p* < 0.001

Host species differed significantly in terms of parasite abundance (GLM: Wilk’s = 0.798, *F* = 10.8, df = 5.213; *p* < 0.001). As compared to *M. blythii*, *M. myotis* had more parasites of the following three taxa: Macronyssidae (*F* = 16.1, *p* < 0.0001), *S. myoti* (*F* = 15.7, *p* = 0.0001), and *N. vexata* (*F* = 25.2, *p* < 0.0001). No significant differences were found in the abundance of *P. dufourii* (*F* = 1.27, *p* = 0.261) and *N. latreillii* (*F* = 0.15, *p* = 0.70) (Fig. 2).

#### Effects of demographic factors, body condition, and hibernation seasons on ectoparasite distribution

GLM revealed only near-significant effects of two ectoparasite species, *S. myoti* (*p* = 0.06) and *N. latreillii* (*p* = 0.068), on the host body condition, and only for one host species (*M. myotis*). In this case, BCI tended to decrease with increasing parasitic abundance (Table 2).

**Table 2:** Results of separate GLM analyses for each ectoparasite and host species

	Macronyssidae			*Spinturnix myoti*		*Nycteribia latreillii*		*Penicillidia dufourii*		*Nycteribia vexata*	
*Myotis myotis*	df	F	*p* value	F	*p* value	*p* value	*p* value	F	*p* value	F	*p* value
BCI [g/mm]	1	0.29	0.591	3.60	0.060	3.39	0.068	0.81	0.371	0.02	0.901
Hg [g/dL]	1	0.00	1.000	1.59	0.210	10.28	0.002**	0.01	0.940	0.54	0.465
Period	1	5.43	0.022*	13.63	0.0004***	6.25	0.014*	12.16	0.001**	2.88	0.092
Sex	1	2.15	0.145	14.63	0.0002***	15.82	0.0001***	16.39	0.0001***	8.86	0.004**
Age	1	1.58	0.212	5.14	0.025*	10.92	0.001***	14.41	0.0002***	14.67	0.0001***
Period × sex	1	0.05	0.816	5.60	0.020*	1.74	0.190	1.28	0.260	1.26	0.264
Period × age	1	1.30	0.257	0.07	0.791	3.81	0.053.	1.75	0.189	0.41	0.522
Sex × age	1	0.57	0.451	1.00	0.319	4.45	0.037*	4.85	0.030*	8.87	0.004**
Period × sex × age	1	0.23	0.631	0.81	0.371	1.19	0.278	0.48	0.488	2.80	0.097
Error	107										
Myotis blythii	df	F	*p* value	F	*p* value	F	*p* value	F	*p* value	F	*p* value
Bci [g/mm]	1	0.24	0.625	0.04	0.841	0.01	0.935	0.08	0.781	0.220	0.640
Hg [g/dL]	1	5.11	0.026*	3.78	0.055.	4.03	0.048*	5.09	0.027*	0.464	0.498
Period	1	2.87	0.094	1.48	0.226	0.03	0.871	0.11	0.741	0.603	0.440
Sex	1	1.83	0.179	12.47	0.001**	4.53	0.036*	14.58	0.0001***	1.964	0.165
Age	1	1.14	0.288	5.39	0.023*	0.80	0.374	0.00	0.945	1.226	0.271
Period × sex	1	0.31	0.576	0.58	0.448	0.43	0.513	0.02	0.888	1.920	0.169
Period × age	1	0.17	0.684	0.20	0.652	0.84	0.363	0.38	0.539	0.562	0.455
Sex × age	1	4.28	0.041*	1.73	0.192	0.05	0.819	0.13	0.723	0.080	0.779
Period × sex × age	1	1.06	0.306	0.01	0.912	1.86	0.176	0.27	0.602	0.004	0.948
Error	90										

Period: December, April; sex: male, female; age: juvenile, adult

*BCI* body condition index, *Hg* hemoglobin concentration

Significant differences are marked with an asterisk: *p < 0.05; **p < 0.01; ***p < 0.001

In *M. myotis*, a significant correlation between ectoparasite abundance and hemoglobin concentration was revealed only for *N. latreillii* (*p* = 0.002), while in *M. blythii*, such a correlation was found for Macronyssidae (*p* = 0.026), *N. latreillii* (*p* = 0.048), and *P. dufourii* (*p* = 0.027) and a nearly significant one for *S. myoti* (*p* = 0.055) (Table 2). In the case of all significant correlations, hemoglobin concentration tended to decrease with increasing parasitic abundance.

The hibernation period affected the abundance of four investigated ectoparasite taxa parasitizing *M. myotis*: Macronyssidae were more numerous in April (*p* = 0.022), while the remaining species in December (*p* = 0.0004 for *S. myoti*, *p* = 0.014 for N. *latreillii*, and *p* = 0.0007 for *P. dufourii*). No effect was found for *M. blythii. M. myotis* females were more heavily parasitized by all summer ectoparasite species: *S. myoti* (*p* = 0.0002), N. *latreillii* (*p* = 0.0001), *N. vexata* (*p* = 0.004), and *P. dufourii* (*p* = 0.0001), but not by Macronyssidae (*p* = 0.145), in which case both host sexes were parasitized to a comparable degree. A similar pattern was observed in *M. blythii* with one exception; no differences were found in *N. vexata*.

Host age affected the abundance of all summer ectoparasite species in *M. myotis*: adult hosts were more heavily parasitized than were juveniles by *S. myoti* (*p* = 0.025), *N. latreillii* (*p* = 0.0013), *P. dufourii* (*p* = 0.0002), and *N. vexata* (*p* = 0.0002). In turn, in the case of *M. blythii*, significantly more ectoparasites on adult bats were found only for *S. myoti* (*p* = 0.023).

A significant two-way interaction between hibernation period and sex was found for *S. myoti* (*p* = 0.020), and a near-significant one for *N. latreillii*, parasitizing *M. myotis* (*p* = 0.053). The interaction between sex and age was significant for all Nycteribiidae species, which parasitized *M. myotis*: *N. latreillii* (*p* = 0.037), *P. dufourii* (*p* = 0.03), and *N. vexata* (*p* = 0.004), but only for Macronyssidae (*p* = 0.041) in *M. blythii*. No significant three-way interactions were identified.

## Discussion

### Host species-specific patterns in ectoparasite abundance and body condition

Although the investigated host species were similar in body size (Arlettaz 1995), thermoregulatory behavior (Schuller et al. 2010), and geographical range and shared both breeding and hibernation shelters (Güttinger et al. 2001), they differed in terms of parasite load. Among the studied summer and winter ectoparasite species, three were more abundant on *M. myotis* than on *M. blythii*. Another two species, *P. dufourii* and *N. latreillii*, which were equally numerous on both bat species, exhibited host species-specific patterns of abundance. Species-specific differences are known primarily from the breeding period: in mixed maternity colonies, *M. myotis* are reported to be significantly more heavily infected by the wing mite *S. myoti* than *M. blythii* (Christe et al. 2003). The other ectoparasite species which often parasitize large Myotis species are known almost exclusively from single-species summer colonies of either *M. myotis* (Hůrka 1964; Zahn and Rupp 2004) or *M. blythii* (Sharifi et al. 2008); hence, in those cases, interspecific differences in parasite abundance remain undetermined. Closely related hosts, such as sibling or sister species, are typically infected by the same ectoparasite species (Mouillot et al. 2006), but they can differ in their abundance (Freeland 1983). Differences may originate mainly from the capacity of the host to deter and/or resist the parasites (Christe et al. 2003; Thomas et al. 2000), or to some extent, from host abundance (Mlynarek et al. 2012; Stanko et al. 2002). Since the two bat species form mixed aggregations during both breeding and wintering (Güttinger et al. 2001), the influence of host availability on parasite abundance is unlikely. While starved or energy-deprived hosts may be less resistant to parasites (Valera et al. 2004; Krasnov et al. 2005a; Hawlena et al. 2005), hosts in good condition can be a better source or nourishment (Christe et al. 2003; Rueesch et al. 2012). If the host’s resources were predictive of ectoparasite abundance, interspecific differences in body condition could explain the differences, but our results do not support this hypothesis. The deterioration of host body condition during hibernation differed to some extent between the host species, but the underlying causes were of intraspecific rather than interspecific nature. During natural hibernation, some bats can actively control their energy balance, primarily by winter feeding (Ransome 1990). This applies almost exclusively to bat species with a gleaning foraging strategy, whose prey (Diptera, Lepidoptera) may be active during warmer days in the winter (Park et al. 2000; Hope et al. 2014). Therefore, differences in the pattern of BCI decline between sex-age host groups can be attributable to the availability of active insects during wintering, as well as to mating behavior; however, this issue is not clear and requires further study.

The preference for one host species both of summer and winter ectoparasites suggests that they have similar mechanisms for selecting an optimum host species. While blood may contain chemical signals useful for identifying the appropriate host species by ectoparasites (Christe et al. 2000; Klein 2005), during hibernation, endocrine secretion is strongly reduced, hormone levels in the blood are marginal (Kawamoto 2003), and the immune function is depressed (Burton and Reichman 1999; Bouma et al. 2012). Alternatively, a species-specific odor-profile may be used by ectoparasites for host selection (Lourenço and Palmeirim 2008; Chaisson and Hallem 2012). The diet of *M. myotis* mainly consists of Carabidae, whose abdominal defensive glands secrete esters (Lečić et al. 2014), while the diet of *M. blythii* is almost exclusively limited to Orthoptera, which do not have chemical defenses, and the resulting differences in odor between the two bat species are strong enough to be noted by humans (indeed, they were observed by the researchers conducting this study). Finally, despite the fact that *M. myotis* was parasitized much more heavily than *M. blythii* was, no parasite-dependent differences in body condition or its decline during hibernation were identified.

The other parameter of body condition, hemoglobin concentration, was slightly lower at the end of hibernation, with the most significant decrease found in first-year females in both host species, but without a species-specific pattern. Despite diminished erythropoiesis during hibernation, bats were characterized by a rather constant level of hemoglobin concentration throughout the year (Grundboeck and Krzanowski 1957; Wołk and Ruprecht 1988). This lack of apparent differences is probably attributable to the storage of red blood cells in the spleen, which is twice as large in inactive animals as in flying ones (Krutzsch and Hughes 1958; Jürgens et al. 1981; Neuweiler 2000). In mammals, hematological parameters such as hemoglobin concentration and hematocrit depend to some extent on sex and age: adult males exhibit higher values than females do, while in juveniles, sex differences are not found (Sealander 1964; Murphy 2014). In turn, juveniles are more sensitive to blood loss than adults are (Hawlena et al. 2007). In this study, first-year females of both host species were more parasitized than males were by the summer ectoparasites, including nycteribiids and wing mites, which are not resistant to starvation (Hůrka 1980; Deunff and Beaucournu 1981), and also feed during winter, but not as frequently as during the summer activity of the host. Therefore, the volume of host blood obtained by these ectoparasites should be higher in females than in males, and in conjunction with reduced hematopoiesis during hibernation, may explain the differences in hemoglobin concentration between first-year males and females.

### Intraspecific factors affect ectoparasite abundance

The body condition parameters of the hosts are correlated with ectoparasite abundance in different ways. We found no clear evidence for ectoparasite load to reduce body condition. Ectoparasites feed mainly on blood and lymph and do not directly use the fat resources of the hosts, and so they have no directional impact on BCI. During hibernation, fat resources are mainly affected by arousals, which constitute the main cost of hibernation (Speakman and Thomas 2003). No significant effect of sex (Park et al. 2000), or even age and BCI, on the frequency of arousals or bouts of torpor has been observed (Jonasson and Willis 2012). Then, the only way in which ectoparasites could influence BCI would be by increasing arousal frequency, which is unlikely. In turn, in this study, hemoglobin concentration was sensitive to ectoparasite abundance and decreased with increasing parasite load, with the only exception being *N. vexata*. A directional impact of ectoparasites on the host’s blood parameters is rarely found (Christe et al. 2000; Hawlena et al. 2006), probably as a result of the rapid production of new cells in response to blood loss (regenerative anemia, Pfäffle et al. 2009); however, it has been observed for hemoglobin concentration (O’Brien et al. 2001; Carleton 2008). Despite the lower abundance of parasites in *M. blythii*, this species exhibited a greater hemoglobin decline and for a greater number of ectoparasite species than in the case of *M. myotis*. These results demonstrate not only an ectoparasite-specific effect on the host, but also one that is independent of the mode of parasite transmission. It can be inferred that ectoparasites prefer hosts which are less sensitive to their direct impact (blood intake), but with independence from the host’s resources allowed survival of temporary food shortage.

During hibernation, the abundance of almost all summer ectoparasites decreased while that of Macronyssidae increased; however, this difference was found only for one host (*M. myotis*). The lack of a significant difference in *M. blythii* may have resulted from a lower load and larger abundance variation of ectoparasites. The summer ectoparasite species feed on blood and they are not entirely deprived of food during hibernation, but its availability can be limited to short episodes during host arousals. In *M. myotis*, torpor bouts last from a 3 to 20 days (Harmata 1987) throughout the hibernation season, which lasts 4–5 months (Wojciechowski et al. 2007) during which up to several dozen arousals occur. Therefore, arousals allow ectoparasites to survive winter, but hibernation contributes to some extent to reducing their abundance. The seasonal changes in ectoparasite abundance were somewhat influenced by host sex and age, but only in the case of *M. myotis*. The abundance of *S. myoti* was found to decrease in females, while in males, it remained at the same level. In turn, in *N. latreilli*, a larger decrease in abundance occurred in adults, while in juveniles, it remained unchanged. The fact that in both cases ectoparasite abundance declined in the sex and age host categories characterized by better body condition may indicate a potential trade-off between the choice of a better host and its stronger defenses. However, the ways in which hosts could bring about reduced ectoparasite abundance during a period of inactivity requires further research. In turn, Macronyssidae can not only feed during hibernation but also reproduce (Dusbábek 1972, Haitlinger 1978), and hence their abundance is higher at the end of hibernation.

The host sex-biased preferences of ectoparasites reproducing in the summer (nycteribiids and wing mites) were distinct from those found in Macronyssidae, which mostly reproduce in the winter. The higher summer parasite abundance in female adult hosts at the beginning of hibernation may result from their larger resources, suggesting a better quality or more profitable host (Christe et al. 2007), and also from other factors, such as age and the resulting greater number of opportunities to encounter parasites (Tinsley 1989; Hawlena et al. 2006). In contrast to previous results (Christe et al. 2007), at the beginning of hibernation, we found sex-biased ectoparasite preferences also among first-year old bats: females were more heavily infected than males were. During the reproduction of summer ectoparasite species, host aggregations (breeding colonies) initially consist almost exclusively of adult females, and later on also of their offspring. Therefore, the possibility of reinfection seems to be a major factor in the female bias of most summer bat ectoparasites. Transfer to the host breeding colony, where ectoparasites also reproduce, is possible almost only by females (whether juvenile or adult) (Webber et al. 2015). In turn, at the end of the hibernation period, there was no apparent difference in the abundance of Macronyssidae between hosts of different sexes and ages. The lack of sex-biased preferences in winter-reproducing ectoparasites is again linked to host availability and may result from the fact that in the wintering site, all sex-age host groups were equally accessible. These results lead to the conclusion that the possibility of reinfection in conjunction with host availability can promote sex-biased parasite infestation in bats.

However, in some cases, sex bias depended on the age of the host. While both male and female juvenile bats were parasitized to a comparable degree, the host sex preferences of ectoparasites differed for adult bats, but only in the case of Nycteribiidae parasitizing *M. myotis* and Macronyssidae parasitizing *M. blythii*. Newborn hosts are more heavily parasitized than adult females aew, but throughout the nursery period, parasite abundance decreases (Encarnação et al. 2012; Christe et al. 2000). These changes can be the effect of acquiring the ability to groom (Giorgi et al. 2001); however, grooming efficiency depends on ectoparasite microhabitat specialization (naked vs. haired skin) (Godinho et al. 2013). Therefore, the lower ectoparasite abundance in juvenile bats is rather caused by the time to encountering parasites than by grooming or an immunocompetence effect (Tinsley 1989; Hawlena et al. 2006).

In conclusion, we found no species-specific patterns in terms of deteriorating host body condition or decreasing hemoglobin concentration during hibernation, despite the differences in the abundance of both summer and winter ectoparasites. While hemoglobin concentration showed a similar pattern, we noted unusual most decreasing in juvenile females in both of host’s species. Since first-year female hosts were more heavily parasitized than males were, it is possible that a significant decrease in hemoglobin content was due to a larger volume of food (blood) consumed by the parasites. However, this does not apply to adult bats.

In turn, at the intraspecific level, ectoparasite abundance had a significant effect on hemoglobin content, but this applied almost exclusively to one host species, *M. blythii*, despite its lower parasite load. On the other hand, body condition was not affected by ectoparasite abundance. This indicates that ectoparasites preferred hosts, which were less sensitive to their activity but did not show a bias for host resources. A better health status may have been preferred due to a higher likelihood of survival until the spring migration. Finally, female-biased parasitism in summer ectoparasite species seems to constitute a strategy that facilitates reinfection in the maternity colony of the host rather than prioritize female resources or survival likelihood. This is consistent with the absence of host sex preferences in winter ectoparasites, as in that case where both host sexes are equally available during the breeding of parasites.
